# New Perspectives on the Potential Role of Aquaporins (AQPs) in the Physiology of Inflammation

**DOI:** 10.3389/fphys.2018.00101

**Published:** 2018-02-16

**Authors:** Rosaria Meli, Claudio Pirozzi, Alessandra Pelagalli

**Affiliations:** ^1^Department of Pharmacy, University of Naples Federico II, Naples, Italy; ^2^Department of Advanced Biomedical Sciences, University of Naples Federico II, Naples, Italy; ^3^Institute of Biostructure and Bioimaging, National Research Council (CNR), Naples, Italy

**Keywords:** aquaporins (AQPs), inflammation, homeostasis, inflammatory diseases, animals

## Abstract

Aquaporins (AQPs) are emerging, in the last few decades, as critical proteins regulating water fluid homeostasis in cells involved in inflammation. AQPs represent a family of ubiquitous membrane channels that regulate osmotically water flux in various tissues and sometimes the transport of small solutes, including glycerol. Extensive data indicate that AQPs, working as water channel proteins, regulate not only cell migration, but also common events essential for inflammatory response. The involvement of AQPs in several inflammatory processes, as demonstrated by their dysregulation both in human and animal diseases, identifies their new role in protection and response to different noxious stimuli, including bacterial infection. This contribution could represent a new key to clarify the dilemma of host-pathogen communications, and opens up new scenarios regarding the investigation of the modulation of specific AQPs, as target for new pharmacological therapies. This review provides updated information on the underlying mechanisms of AQPs in the regulation of inflammatory responses in mammals and discusses the broad spectrum of options that can be tailored for different diseases and their pharmacological treatment.

## Introduction

The inflammatory process and the complex mechanisms governing its regulation have always attracted scientific interest, focusing on the role of the various parties involved. Inflammation is a multifaceted phenomenon involving the body's physiological response to injury. The inflammatory tissue damage can be determined, among others, by trauma, heat, chemicals, toxins, and microbes. Both acute and chronic inflammatory process implicates an enormous expenditure of metabolic energy, loss of function, tissue damage, and destruction, involving different immune cells. Many signals orchestrate all inflammatory responses and a pivotal role is played by the immune system. Generally, it can be divided into two interconnected subsystems. The innate or non-specific immune system includes cells and processes that protect the host from infections by pathogen organisms, detecting and signaling the occurrence of infection (Takeda et al., 2003; Medzhitov, 2007). These signals are necessary to trigger the inflammatory cascade and to activate the adaptive immune response, the second subsystem. During activation, numerous substances released by the injured tissues induce alterations to the surrounding uninjured tissues. Simultaneously, cells of hematopoietic origin are recruited to damaged sites in order to resolve the inflammation and initiate tissue repair. Several of the products released from these cells, differing in origin and composition, constitute the real messengers of the inflammation process, and have been often viewed as drug targets.

It is interesting to examine the mechanisms and the mediators underlying the initiation of inflammation starting by the alteration of cellular and tissue homeostasis: an important role is played by the membranes that contribute to maintain the equilibrium in the microenvironment. Indeed, the cells normally modulate their internal environment in response to external changes; the loss of their ability to regulate fluid movement through the membrane lead to severe alteration of cell physiology (Loo et al., 1996). This cellular disorder affects, as a direct consequence, numerous biochemical processes, including alterations of protein structure and function and hence enzyme activity (Brocker et al., 2012). During homeostasis perturbation, as in conditions characterized by local or systemic inflammation, disease susceptibility appears and this phenomenon is associated with abnormal ion transport (Kotas and Medzhitov, 2015). In addition, many inflammatory signals, including cytokines and chemokines, can modify tissue/cell homeostasis by changing their sensitivity to homeostatic signals or by modifying gate or channel access (Medzhitov, 2008). The swelling of cells and tissues with a surplus of extracellular fluids (edema), is a clear sign of homeostasis disturbance and inflammation.

Aquaporins (AQPs) represent a new class of proteins extensively distributed on cell membrane that form pores and primarily act in several transporting and trafficking processes. Growing data have addressed their possible involvement in inflammation, participating as regulators of innate host defense at cell membrane level. AQPs could be involved in inflammasome activation regulating cell volume (Compan et al., 2012). Indeed, NALP3 inflammasome activation is induced by sodium overload and water influx, both features of cell swelling (Schorn et al., 2011), that represents a clear inflammatory response involving pro-inflammatory cytokine synthesis.

Here, we report recent data on the potential role of AQPs in the inflammatory process, as an adaptive response to the loss of cellular homeostasis or tissue damage. In addition, the function or alteration of AQPs in different inflammatory diseases or animal models is also discussed.

## Aquaporins

**Figure 1:**
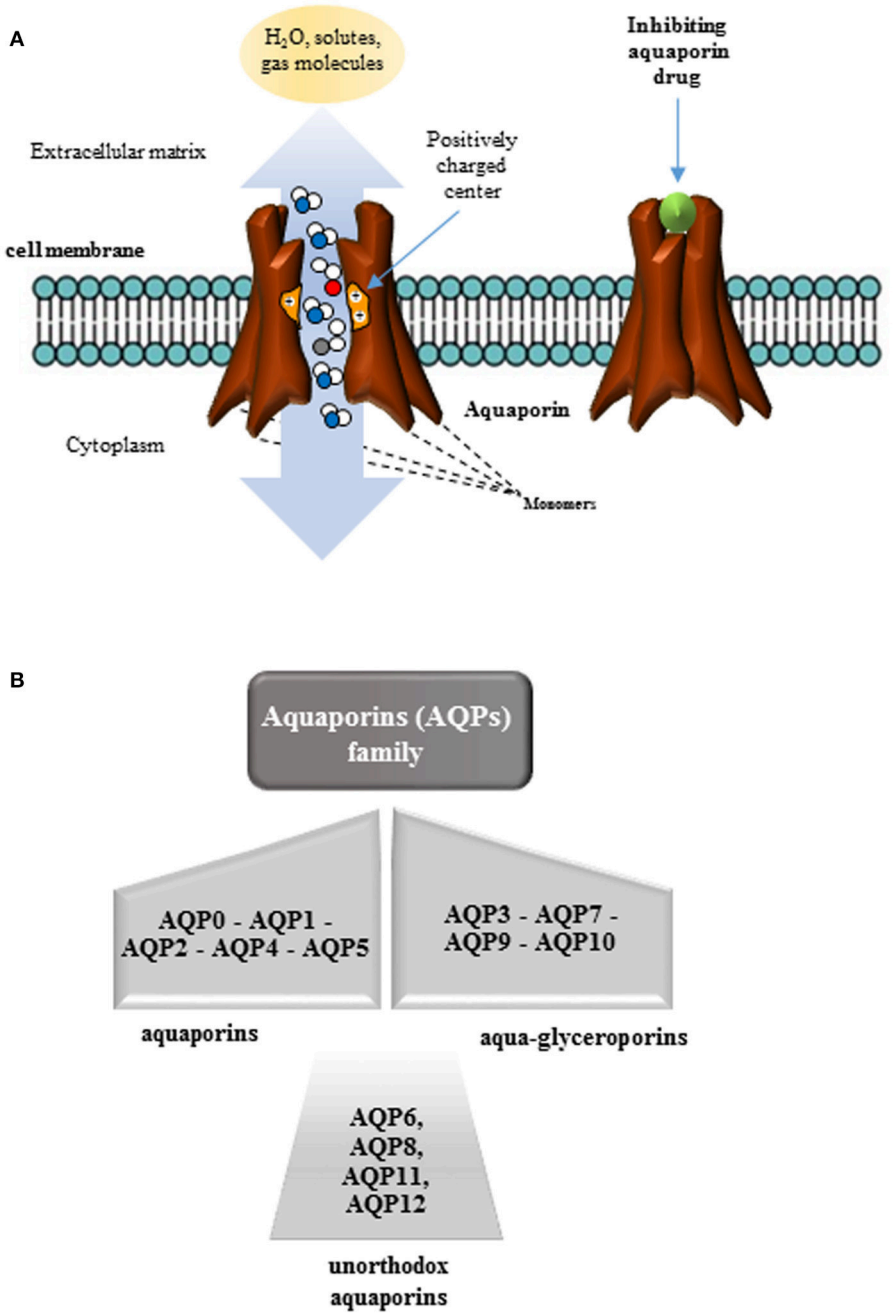
Organization of aquaporins (AQPs). **(A)** AQPs are proteins structurally organized in monomers that assemble in a tetrameric structure in membranes to form a pore. AQPs, as membrane channels, facilitate the transport of water and small neutral solutes across biological membranes of most living organisms depending on the size of the pore. Several stimuli or aquaporin inhibiting drugs, can act closing these channels. **(B)** The aquaporin family is constituted by 13 water channel proteins in mammals (AQP0–AQP12) subdivided into three classes: (i) aquaporins transporting exclusively water; (ii) aquaglyceroporins transporting not only water but also small solutes, and (iii) unorthodox aquaporins with a yet unknown function, poor water permeability but permeable to other small uncharged solutes.

AQPs constitute a group of integral membrane proteins characterized by six transmembrane helices, that are organized in monomers, dimers, and tetramers, forming pores in the membrane of biological cells (Verkman, 2013). The first aquaporin discovered, initially called CHIP28 and later renamed as Aquaporin (AQP)1, was isolated in human erythrocytes (Denker et al., 1988; Preston and Agre, 1991) and in renal proximal tubule membranes. Since this discovery, many other proteins belonging to the same aquaporin family were described not only in mammalian cells, but also in all kingdoms of life, including bacteria (Kayingo et al., 2001), plants (Maurel et al., 2008), and fungi (Pettersson et al., 2005). Forming pores at the level of biological membranes, AQPs act as selective channels allowing the water transportation (aquaporins) and small molecules or solutes (aquaglyceroporins) (Agre et al., 1998) (Figures 1A,B). In the great family of aquaporins constituted by 13 proteins, a newly group named unhortodox aquaporins has been defined (Ishibashi et al., 2011). These last proteins are less understood and, in part, differ from the other groups for their structure and subcellular distribution. The widely distribution of AQPs in cells and tissues has increased the scientific interest toward their structural and functional characterization contributing to strength the idea that the water permeability is required for a variety of physiological processes. This observation is based on the consideration that water constitutes the major component of a living organism and that continuous exchange of water takes place in almost all body-tissues.

Accordingly, many data demonstrate that AQPs show a role in maintaining the homeostasis of many physiological processes related to secretive and absorptive activities of several tissues, such as kidney, salivary gland, lung, skin, sweat glands, and intestine (Laforenza, 2012; Pelagalli et al., 2016b). Moreover, these proteins participate in maintaining a constant water homeostasis in whole organism as essential prerequisite for their life. More recently, emerging evidence have confirmed that other cellular processes are controlled by AQPs, including cell adhesion, signaling, volume regulation and protein expression (Kitchen et al., 2015).

Moreover, the ability of these channel proteins to regulate cell volume, as well as cell migration and apoptosis, all related to the inflammation process, makes them useful tools to investigate their relevant role in physiological and pathological status.

## Aquaporins and inflammation

Immune system and host-pathogen communication work together in host-pathogen interplay.

In particular, cells involved in inflammation can undergo modifications of osmotic microenvironment causing an increase in cell hydraulic permeability and size and thus, alterations in cytoskeletal structure (Maidhof et al., 2014). In some cases, weak bacterial stimuli can induce tissue impairment, but also a more intense response and damage that results in a chronic inflammation.

Many articles have highlighted the possible involvement of AQPs in the development of inflammatory processes also considering that several of them are expressed in cells of innate and adaptive immunity (Ishibashi et al., 1998; Moon et al., 2004). In particular, AQPs are involved in the phagocytic functions, and also in specific processes related to immune cells (i.e., activation and migration) (De Baey and Lanzavecchia, 2000; Jablonski et al., 2004; Zhu et al., 2011; Rabolli et al., 2014).

### Aquaporins and phagocytic functions of immune cells

Cell volume and shape modifications of macrophages promptly occur during phagosome development (Clarke et al., 2010; Tollis et al., 2010), modulating water transport and volume regulation necessary for phagocytic cup formation. The participation of AQP3 in phagosome formation has been proposed (Zhu et al., 2011), postulating a mechanism similar to that observed in immature human dendritic cells (De Baey and Lanzavecchia, 2000). This mechanism identifies the glycerol transport as a key element for macrophage phagocytosis. Indeed, in a model of bacterial peritonitis a greater mortality in AQP3-deficient mice was observed respect to wild-type mice (Zhu et al., 2011). In peritoneal macrophages obtained from AQP3(^−^/^−^) mice, water and glycerol permeability was reduced compared to those obtained from AQP3(^+^/^+^) mice. Moreover, glycerol supplementation partially recovered the ATP content and the impairment of macrophage function in AQP3(^−^/^−^) mice. AQP3 was identified as a novel key element in macrophage immune function, facilitating water and glycerol transportation, and its subsequent participation in phagocytic and migration activity. A crosstalk in glycerol and glucose metabolic pathways was also evidenced in AQP3 effects. Similar data for glucose and glycerol content modification were also observed in AQP3 (^−^/^−^) keratinocytes (Hara-Chikuma and Verkman, 2008), identifying a possible new role for glycerol in macrophage energy metabolism.

Recent evidence has indicated an innovative AQP9 role for bacteria (*Pseudomonas aeruginosa*)–macrophage communication and for the sensing system in this process (Holm et al., 2015). In particular, it has been demonstrated that macrophages presenting AQP9 expression and infected by *P. aeruginosa* undergo modification of AQP9, and subsequent water fluxes, affect their shape and protrusive activity. These results confirm the role of AQP9 in macrophages during infection, clarifying how these proteins, participating as mediators to relationship between bacteria and macrophages, can affect the development of infection, inflammation, and the progression of the disease.

### Aquaporin involvement in migration of immune cells

The first demonstration of AQPs involvement in cell migration was reported by Loitto et al. (2002) indicating an impaired neutrophil migration after AQP9 blockage. Subsequently, other studies confirmed the AQP role in cell migration, showing AQP1 and AQP4 localization at the leading edge in migrating CHO cells and astroglial cells, respectively (Saadoun et al., 2005a,b). Other data have largely demonstrated that several AQPs facilitate migration of immune cells (Papadopoulos et al., 2008). In particular, chemokine-dependent T cell migration requires AQP3-mediated hydrogen peroxide uptake (Hara-Chikuma et al., 2012), regulating downstream intracellular signaling in cutaneous immune response (Miller et al., 2010). AQP3 is also expressed in macrophages (Zhu et al., 2011) and is regulated by several factors and conditions (TNFα, PPARγ, calcium, and low pH) (Horie et al., 2009; Jiang et al., 2011). These results demonstrate AQP3 involvement in the inflammatory process.

More recently, a study focusing on AQP1 has demonstrated its effect on macrophage migration, suggesting that some phenotypic and migratory modifications of these cells may be regulated by this water channel that results crucial for the switch of M0/M2 phenotype (Tyteca et al., 2015).

## Potential role of aquaporins in different models of inflammation

### Potential role of aquaporins in models of lung injury and inflammation

Numerous evidence clearly demonstrates that the mammalian lung expresses at least three AQPs whose role in lung damage or inflammation has been in part investigated (Table 1).

**Table 1:** Summary of studies illustrating the possible involvement of AQPs in animal experimental models of inflammatory-based diseases.

**Animal species**	**Inflammation type**	**Method for induction**	**AQP analyzed**	**Methods adopted for the AQPs determination**	**References**
Rat	lung injury	LPS	AQP1	IHC, WB	Li et al., 2007
Rat	lung injury	Ventilation	AQP1, AQP5	mRNA expression, WB	Fabregat et al., 2016
Rat	lung injury	Hyperoxia	AQP5	IHC, mRNA expression	Tan et al., 2006
Mouse	lung injury	LPS (5mg/kg); HCl (0.1 N); Ventilation	AQP1, AQP4, AQP5, AQP9	RT-PCR, WB	Vassiliou et al., 2017
Mouse	lung injury	LPS	AQP1, AQP3, AQP4, AQP5	IHC, RT-PCR, WB	Li et al., 2008
Rat	brain edema	Hypoxia	AQP4	mRNA expression, WB	Song et al., 2016
Rat	neuromyelitis optica	IgGAQP4^+^	AQP4	IHC, TEM, WB	Marignier et al., 2016
Mouse	Parkinson disease	MPTP/probenecid PD models	AQP4	IHC, mRNA expression, WB, biochemical assays	Sun et al., 2016
Rat	Colitis	TNBS (2.5 mg/ml in 50% methanol) (7 days)	AQP3, AQP8	IHC, mRNA expression, WB	Zhao et al., 2014
Mouse	Colitis	TNBS, DSS, CD4CD45RB transfer	AQP8	microarrays	Te Velde et al., 2008
Mouse	Colitis	DSS (2.5%) drinking water (7 days)	AQP4, AQP7, AQP8 (colon)	IHC, mRNA expression, WB	Hardin et al., 2004
Mouse	Colitis	DSS (4%) drinking water (8 days)	AQP4 (caecum)	RT-PCR, microarray-IF	Hansen et al., 2009
Mouse	Colitis	DSS (6%) drinking water (4 days)	AQP3	IHC, WB, biochemical assays	Thiagarajah et al., 2007
Mouse	Diarrhea	5-FU (50 mg/kg) (4 days)	AQP4, AQP8	RT-PCR, WB	Sakai et al., 2013
Mouse	Diarrhea	MgSO_4_	AQP2, AQP3	IHC, WB	Liu et al., 2014
Rat	Osteoarthritis	Anterior cruciate ligament and medial collateral ligament resection	AQP1	mRNA expression, RT-PCR,	Gao et al., 2011
Rat	Osteoarthritis	Meniscus resection	AQP1	IHC, RT-PCR, biochemical assays	Fujitsuka et al., 2015

*DSS, Dextran sodium sulfate; LPS, lipopolysaccharide; TNBS, 2,4,6-trinitrobenzene sulphonic acid; 5-FU, 5-floruracil; IHC, Immunohistochemistry; MPTP, 1-methyl-4-phenyl-1,2,3,6-tetrahydropyridine; PD, Parkinson disease; RT-PCR, real time-PCR; TEM, transmission electron microscopy; WB, western blotting*.

In particular, AQP1 is expressed in microvascular endothelia and pneumocytes (Nielsen et al., 1993; Folkesson et al., 1994). AQP4 and AQP5 were detected in airway and alveolar epithelial cells, respectively (Nielsen et al., 1997) and their distribution and physiological role has been reviewed in lung (Verkman et al., 2000). Thereafter, Liu et al. (2007) evidenced AQP3 expression in healthy and cancer lung, demonstrating that this AQP is extensively expressed in respiratory tract regulating water homeostasis. AQP3 seem to be implicated into tumor differentiation and processes related to clinical stage in lung adenocarcinomas (Liu et al., 2007). On this regard, the specific distribution of various AQPs in lung adenocarcinoma (AQP1, AQP3, and AQP5) has suggested that these proteins could be involved in different and distinct aspects of the cancerous process. In particular: (1) AQP1 localized on lung capillaries could be involved in the development of angiogenesis; (2) AQP3 could participate in several regulatory pathways, while (3) AQP5 could promote cell proliferation and tumor invasion (Wang et al., 2015).

All the data available demonstrate an interesting contribution given by lung AQPs in regulating fluid trafficking between the air space and cellular, interstitial or vascular compartments. In addition, evidence shows that expression of AQPs is modulated by growth factors, inflammatory mediators, and osmotic stress in the respiratory physiology (King et al., 1997; Borok et al., 1998; Towne et al., 2000).

However, after pulmonary infection, numerous processes altering the lung physiology occur (Peteranderl et al., 2017). In particular, autocrine and/or paracrine mediators cause several pathophysiological modifications of the alveolar–capillary barrier and of epithelial ion channel and pump expression altering vectorial ion gradient. Among these mediators, pro-inflammatory cytokines (TGF-β, TNFα, interferons, IL-1β) are released after infection by different bacteria (i.e., *Streptococcus pneumoniae, Klebsiella pneumoniae*, or *Mycoplasma pneumonia*) or virus. These inflammatory players can induce edema formation and reduce alveolar fluid clearance, modifying the expression of: (i) epithelial Na,K-ATPase, (ii) epithelial ion channels, and (iii) fibrosis membrane conductance regulator (Peteranderl et al., 2017). This alteration can represent also the result of altered gas exchange involved in the modulation of the alveolar–capillary fluid homeostasis modulated by AQP5 and AQP4 (Musa-Aziz et al., 2009) or AQP1 (Al-Samir et al., 2016).

Towne et al. (2000) demonstrated a marked and significant reduction of AQPs (AQP1 and AQP5) in lung directly correlated with an increase in tissue wet-to-dry weight ratios in a model of mice infected with adenovirus. The reduction in AQP1 and AQP5 expressions was noticed at sites distant from that of infection, suggesting a humoral regulation of these AQPs. Albeit, these data did not define the precise activity of AQP1 or AQP5 in infective process, they give an important contribution in the field, confirming AQP involvement in the pathophysiological processes of the respiratory tract (King et al., 2000).

Further data supports these findings showing that Th2 cytokines and IL-4, both involved in mucin gene expression, are down-regulated in AQP5 knockout mouse (Karras et al., 2007), thus suggesting the contribution of this channel protein in effective Th2-driven responses to allergens. More recently, the relationship between the AQP5 deletion and elevated IFN-α and IL-2 production was evidenced, indicating that this protein acts in the shift from Th2 toward Th1 response in a murine model of mucous hyperproduction during antigen-induced airway inflammation (Shen et al., 2011). It is well-known that Th2 cytokines operate in these pathologies with different mechanisms such as eosinophil recruitment, airway hyper-responsiveness and mucus hypersecretion (Tomkinson et al., 2001; Walter et al., 2001).

More recently, AQPs were described as potential downstream targets of altered gene expression in several murine models of induced asthma (by allergen ovalbumin or IL-13) (Krane et al., 2009). AQP3 and AQP5 gene expressions appear as quite early modification in the lung response to mIL-13 induced airway constriction. Indeed, preclinical data showed that AQP3 potentiates allergic airway inflammation in OVA-induced asthma (Ikezoe et al., 2016). A significant reduction of airway inflammation observed in AQP3 deficient mice, respect to wild-type mice, was associated to *in vivo* and *in vitro* results, showing that an increase of chemokines (i.e., CCL24 and CCL22) was induced by AQP3 through a control mechanism of the cellular H_2_O_2_ production in M2 polarized alveolar macrophages (Ikezoe et al., 2016).

### Involvement of aquaporins in neuroinflammation

Accumulating evidence in humans and animals supports physiological and pathological role of AQPs expression and function in the nervous system (Table 1). The potential contribute of these channel proteins in the neuroinflammation has been widely investigated, examining several diseases caused by a failure of innate immunity, such as neuromyelitis optica (NMO) and multiple sclerosis (Oklinski et al., 2016). The channel protein AQP4 is expressed in astrocytes in CNS and regulates the brain water flux, neuroexcitation, and astrocyte migration (Verkman et al., 2006). In fact, lesions observed in NMO patients show that specific autoantibodies targeting AQP4 are expressed on astrocytic membrane and thus, alter cell functions through different mechanisms. Among these, activation of complement, cellular cytotoxicity mediated by an antibody-dependent mechanism, or both mechanisms were evidenced (Bennett et al., 2009; Bradl et al., 2009). AQP4 represents a specific target for NMO-IgG (Fukuda and Badaut, 2012; Hinson et al., 2012). Moreover, it has been clearly established that APQ4 is involved in neuroinflammation (i.e., water intoxication and ischemic stroke), evidencing a reduction of brain edema and swelling of pericapillary astrocytic foot processes in AQP4-deficient mice (Manley et al., 2000). These results indicate a key role for AQP4 in controlling brain water transport, and propose that AQP4 blockage could be represent a new therapeutic strategy for ameliorate conditions of cerebral edema at the basis of several brain pathologies.

Papadopoulos et al. (2008) reviewed evidence that AQPs facilitate cell migration both *in vitro* and *in vivo* in a variety of cell types such as endothelial cells and astrocytes. In particular, AQP1 deletion diminishes endothelial cell migration, limiting tumor angiogenesis and growth, on the other hand, AQP1-expressing tumor cells have enhanced local infiltration and metastatic effects. AQP4 deletion reduces the migration of reactive astrocytes, damaging glial scarring after brain injury. AQPs modulating cell migration can be implicated in several processes such as angiogenesis, tumor metastasis, wound healing, glial scarring, and other events requiring prompt cell movement. The mechanisms by which AQPs act, is probably related to actin polymerization/depolymerization and variation of transmembrane ionic fluxes and osmolality (Papadopoulos et al., 2004). AQPs could thus increase osmotic water flow in cell protrusions of the plasma membrane that shape during cell migration. Indication for involvement of AQPs in a similar process, i.e., secretory vesicle exocytosis, has been previously described (Cho et al., 2002; Sugiya and Matsuki, 2006).

**Figure 2:**
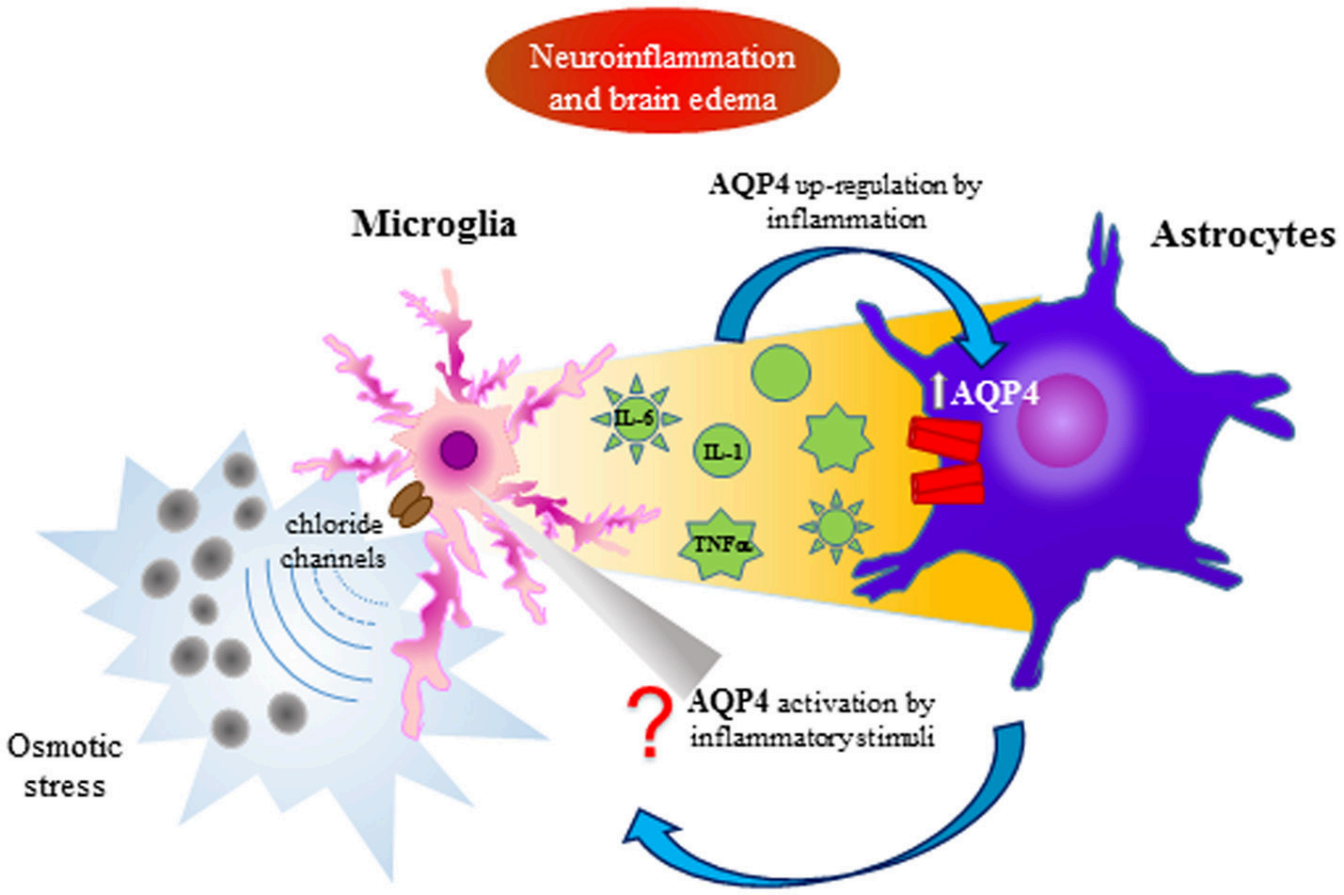
AQP4 involvement in the interplay between astrocytes and microglia during neuroinflammation and brain edema. Microglia activation by osmotic stress induces cytokine release affecting astrocyte activation by the AQP4 up-regulation. A bidirectional communication between astrocytes and microglia can be supposed.

Pro-inflammatory role for AQP4 was confirmed by Li et al. (2011) demonstrating that LPS, administered by intracerebral injection, induced greater neuroinflammation in wild-type than in AQP4-knockout mice, and cytokine (TNFα and IL-6) production was reduced in astrocyte cultures obtained from AQP4-knockout mice. These data suggested that astrocyte swelling and cytokine release are AQP4-dependent in this cell type. In this way AQP4 is involved in the communication between microglia and astrocyte and their functions (Figure 2; Ikeshima-Kataoka, 2016). For all these reasons, the decrease in AQP4 water transport or AQP inhibitors could play a protective role in neuroinflammation, modulating brain edema and cell migration.

### Aquaporins in bowel diseases

AQPs expression and their relevant role in physiological and pathological processes have been evidenced in gastrointestinal tract of human and mammalian species (Laforenza, 2012; Pelagalli et al., 2016b). Gut involvement of AQPs was determined in many mechanisms that mediate water transport (intestinal permeability, and fluid secretion/absorption). Regarding AQPs regulation, it is known that different substances (hormones and dietary components) act modifying their expression and thus alter fluid homeostasis and several local mechanisms (De Luca et al., 2015; Squillacioti et al., 2015; Pelagalli et al., 2016a,b). In this regulation, a role for cAMP was also addressed (Hamabata et al., 2002).

**Figure 3:**
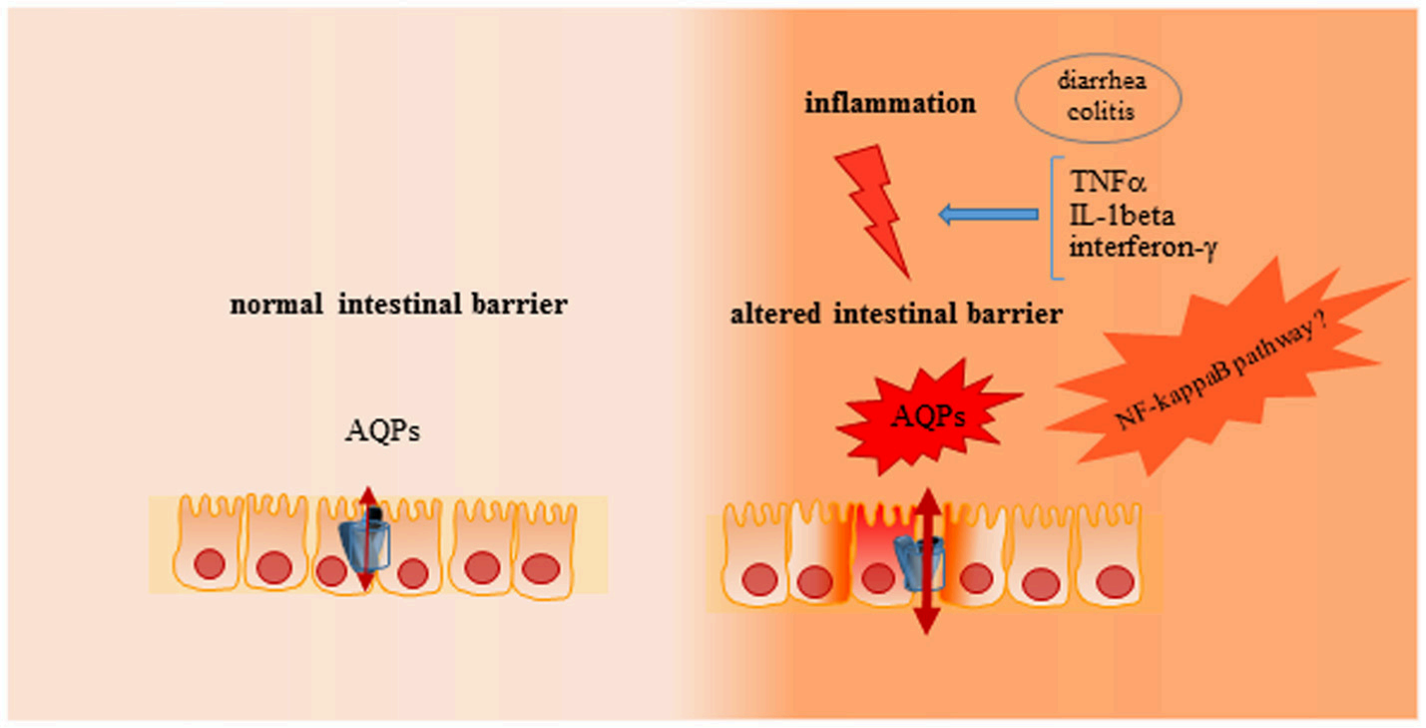
Role of AQPs on intestinal barrier in physiological or inflammatory conditions. In physiological condition an adequate intestinal permeability is present in gut, with a functional epithelial cell proliferation and turnover, characterized by an adequate stress response and prompt epithelial restitution after injury. In inflammatory condition (i.e., colitis or diarrhea) altered stress response is evident, with associated to a reduction of epithelial restitution after injury and dysregulation of electrolytes and water transport mediated by AQPs.

Inflammatory bowel diseases (IBDs) are inflammatory relapsing diseases of gastrointestinal tract with a chronic aberrant stimulation of immune system, gut inflammation and leakage of fluid, solutes, and lipids in bowel mucosa, involving gut microflora (Mayer, 2010). IBDs have been also characterized for a dysregulation in electrolyte and water transport with resultant alteration of permeability and diarrhea (Dunlop et al., 2006; Zhou et al., 2009; Martínez et al., 2012), for this reason the relationship between colitis and AQPs have been extensively investigated (Table 1). In particular, the remarkable increase of intestinal membrane permeability observed in these diseases has suggested the participation of AQPs (Figure 3). Moreover, it is also well-known that cytokines, as important signaling molecules of the intestinal immune system, are correlated to the severity of inflammation (Kim, 2011; Strober and Fuss, 2011). Among them, TNFα and IL-1β play a pivotal role in gut inflammatory processes directly, influencing intestinal epithelial tissue behaving as a frontline between genetic, environmental, and immunological factors (Hering et al., 2012; Keita and Soderholm, 2012).

In 2007, Guttman et al. evidenced for the first time the direct correlation between AQPs and diarrhea, defining AQP contribution to diarrhea caused by attaching and effacing bacteria (i.e., enterohemorrhagic *Escherichia coli* and enteropathogenic *E. coli*) pathogenesis (Guttman et al., 2007). Very recently, Chao and Zhang (2017) evidenced a possible relationship between AQPs (AQP1, AQP3 and AQP8) expression and NF-κB pathway in a model of IBD. Numerous findings indicate NF-κB pathway as the main regulator of several processes (pro-inflammatory cytokine production, leukocyte recruitment, or cell survival), and its involvement in relation to AQPs (Ito et al., 2006; Hasler et al., 2008). Regarding the possible link between TNFα and AQPs, it has been evidenced that TNFα acts modulating AQP3 expression (down- or up-regulation), according to the cell type involved, through different signaling pathways (Tancharoen et al., 2008; Horie et al., 2009). More recently, it was demonstrated that AQP3 downregulation is mediated by the inhibition of constitutive transcriptional activity at the AQP3 promoter in HT-29 cells (Peplowski et al., 2017). In another cell line (CMT93) (Dicay et al., 2015) demonstrated that IFNγ limits epithelial AQP1 expression through the activation JAK/STAT3 pathway. In addition, the authors provided data that demonstrated a role for IRF-2 in the basal expression of AQP1, and that IFNγ was able to regulate AQP1 expression (Dicay et al., 2015).

Colitis, as gut IL-dependent inflammation, is mediated by a Th1 cell response and AQPs affect it, interfering in the proliferative activities of colon epithelial cells. In AQP3 null mice, dextran sodium sulfate (DSS) induced severe colitis, characterized also by hemorrhage in colon, marked epithelial cell loss and death after 3 days, while wild-type mice showed remarkably less severe colitis, surviving to >8 days (Thiagarajah et al., 2007). Moreover, in AQP3 null mice, cell proliferation was greatly reduced. A new role for AQP3 in enterocyte proliferation was likely related to its glycerol-transporting function. In fact, oral glycerol administration largely enhanced survival of AQP3 null mice and reduced the severity of colitis. These data identify AQP3 deficiency as the cause of a reduced AQP3-facilitated glycerol transport, compromising cell metabolism.

Recently, a detailed study focused on the possible role of AQPs in both severe IBDs (Crohn's disease and ulcerative colitis), demonstrating a different distribution of these channel proteins in the gut and the existence of a direct relationship between intestine inflammation and physiological water/solute trafficking and regulation (Ricanek et al., 2015).

### Aquaporins and bone and cartilage diseases

Recently, data on several inflammatory diseases affecting bone and cartilage has involved AQPs. It is well-known that the principal pathological phenomena associated with rheumatoid arthritis (RA) are characterized by enormously elevated levels of inflammatory cytokines secreted by activated B and T cells causing damage of the cartilage and bone. At the same time, different AQPs have been found in cartilage cells where they regulate the traffic of ions and molecules (Mobasheri et al., 2004) and thus, regulate the cartilage physiology.

In particular, Nagahara et al. (2010) evidenced that in synovial tissues from patients with osteoarthritis (OA) and RA, TNFα could regulate either AQP9 mRNA and protein expression (Nagahara et al., 2010). This result has suggested a particular role for cytokines that, altering the activity of glucose transporters, important for chondrocyte metabolism (Mobasheri et al., 2002; Richardson et al., 2003), could influence AQP function. According to this mechanism, AQP1 on chondrocyte membrane could act as regulator of metabolic or extracellular matrix water (Trujillo et al., 2004), suggesting that chondrocytes might respond to changes in their ionic and osmotic environment modifying volume regulatory behavior. The direct involvement of AQPs in the pathogenesis of this disease was investigated in a model of OA cartilage evaluating AQP1 mRNA by RT-PCR and demonstrating that up-regulation of AQP1 was related to the chondrocyte apoptosis (Table 1) (Gao et al., 2011).

Recently, for the first time, Cai et al. (2017), identified AQP4 as potential responsible of RA pathogenesis in adjuvant-induced arthritis (AIA) rat model. The results showed that the reduced mRNA levels of collagen type II and aggrecan, observed in cultured AIA chondrocytes, were reverted by acetazolamide treatment. AQP4 inhibition obtained with acetazolamide promoted extra cellular matrix production of AIA chondrocytes *in vitro* (Cai et al., 2017).

## Inflammatory diseases in domestic animal species: the involvement of aquaporins

In the recent last years, many studies have examined inflammatory diseases in domestic animal species, albeit available data are limited considering several limitations for ethical problem in these species respect to laboratory animals. However, evidence indicate that animals as well as humans can suffer of several inflammatory diseases whose possible mechanisms are not always well-defined. Even if, different diseases have been investigated in domestic species, few are the studies regarding the possible link between AQPs and inflammation. The most investigated species is the dog most likely because it is very similar to humans.

In particular, inflammation-based diseases, in organs and systems, like gut, central nervous system, and lung have been investigated in dog species with the perspective to clarify their pathophysiology finalized to adequate therapeutic protocols. On this regard Cerquetella et al., 2010) studied some particular aspects regarding dysbiosis networks in dog IBDs, evidencing differences and similarities with humans. The results of this study providing new important contributes for translational medicine require further development of scientific research for understanding differences between dog and human in some bacteria species. In addition, an interesting study showed an increase of AQP4 and IL6 levels in cerebrospinal fluid (CSF) of dogs affected by idiopathic communicating internal hydrocephalus and a reduction of these proteins after ventriculo-peritoneal shunting (Schmidt et al., 2016). In addition, a study on acute respiratory distress syndrome in beagle dogs showed a clear inflammatory process characterized by TNFα increase that can facilitate the secretion of cytokines, such as IL-1A, IL-6, and IL-10 (Zhao et al., 2012). Moreover, the decreased AQP1 and AQP5 expression observed as possible consequence of pulmonary capillary membrane barrier damage suggests their possible involvement in the regulation of these fluid trafficking mechanisms along this membrane.

Moreover, in avian species AQPs expression has been investigated at level of nasal gland and its fluid secretion. In particular, AQP1 and AQP5 seem to play a role in modulating nasal fluid secretion that it is always hypertonic, differently from vertebrates. (Müller et al., 2006).

## Conclusions

Described evidence suggest that AQPs are not only simple transporting proteins, since their dysregulation occurs in immune and epithelial cells in response to infectious and inflammatory stimuli.

The discovery of AQPs involvement in inflammation certainly can contribute to the knowledge of the complex mechanisms regulating host-pathogen communications. Overall, it seems clear that AQPs are new possible candidates as therapeutic potential target in modulating edema, cell migration and, inflammatory cytokines and mediators release.

Future studies are needed to better understand the molecular mechanisms driving osmotic stress-induced inflammatory response and to clarify the unravel signaling pathways involved in the regulation of AQPs functions. The acquisition of these basic skills could help to clarify the importance of osmotic imbalances not only in inflammation and inflammation based-diseases but also in cancer.

## Author contributions

RM: provided intellectual input, corrected drafts of the review and refined final version; CP: conceived some aspects of review and corrected its drafts; AP: conceived the review, wrote the original manuscript, corrected several drafts and refined final version.

### Conflict of interest statement

The authors declare that the research was conducted in the absence of any commercial or financial relationships that could be construed as a potential conflict of interest.
